# Increasing proportions of extended-spectrum β-lactamase-producing isolates among *Escherichia coli* from urine and bloodstream infections: results from a nationwide surveillance network, Finland, 2008 to 2019

**DOI:** 10.2807/1560-7917.ES.2023.28.43.2200934

**Published:** 2023-10-26

**Authors:** Heikki Ilmavirta, Jukka Ollgren, Kati Räisänen, Tuure Kinnunen, Antti J Hakanen, Jari Jalava, Outi Lyytikäinen

**Affiliations:** 1 University of Eastern Finland (UEF), Kuopio, Finland; 2 ISLAB Laboratory Centre, Kuopio, Finland; 3 Department of health security, Finnish Institute for Health and Welfare (THL), Helsinki, Finland; 4 Tyks Laboratories, Turku University Hospital (TYKS) and University of Turku (UTU), Turku, Finland

**Keywords:** Escherichia coli, Male, Female, Urinary tract infection, Bacteraemia, Bloodstream infection, Drug Resistance Bacterial, ESBL, Fluoroquinolone, AMR

## Abstract

**Background:**

*Escherichia coli* is the leading cause of urinary tract infections (UTI) and bloodstream infections (BSI), and the emergence of antimicrobial resistance (AMR) in *E. coli* causes concern.

**Aim:**

To investigate changes in the proportion of extended-spectrum β-lactamase (ESBL) producing isolates among *E. coli* isolated from urine and blood in Finland during 2008–2019.

**Methods:**

Susceptibility testing of 1,568,488 urine (90% female, 10% male) and 47,927 blood *E. coli* isolates (61% female, 39% male) from all Finnish clinical microbiology laboratories during 2008–2019 was performed according to guidelines from the Clinical and Laboratory Standard Institute during 2008–2010 and the European Committee on Antimicrobial Susceptibility Testing during 2011–2019. A binomial regression model with log link compared observed trends over time and by age group and sex.

**Results:**

The annual proportion of ESBL-producing *E. coli* isolates among *E. coli* from blood cultures increased from 2.4% (23/966) to 8.6% (190/2,197) among males (average annual increase 7.7%; 95% CI: 4.4–11.0%, p < 0.01) and from 1.6% (28/1,806) to 6.4% (207/3,218) among females (9.3%; 95% CI: 4.8–14.0%, p < 0.01). In urine cultures, the proportion of ESBL-producing *E. coli* isolates increased from 2.2% (239/10,806) to 7.2% (1,098/15,297) among males (8.8%; 95% CI: 6.5–11.3%, p < 0.01) and from 1.0% (1,045/108,390) to 3.1% (3,717/120,671) among females (8.6%; 95% CI: 6.3–11.0%, p < 0.01). A significant increase was observed within most age groups.

**Conclusions:**

Considering the ageing population and their risk of *E. coli* BSI and UTI, the increase in the annual proportions of ESBL-producing *E. coli* is concerning, and these increasing trends should be carefully monitored.

**Key public health message**

**What did you want to address in this study?**

Extended-spectrum β-lactamase (ESBL)-production makes the bacterium *Escherichia coli* resistant to several β-lactam antibiotics. We wanted to investigate whether the proportion of ESBL-producing *E. coli* among urinary tract and bloodstream infections caused by *E. coli* increased in Finland during 2008–2019 and understand the risk these bacteria pose on persons of different sex and age.

**What have we learnt from this study?**

The proportion of ESBL-producing isolates among *E. coli* from urinary tract and bloodstream infections increased in the entire Finnish population by nearly 9% per year and this proportion was highest in males and bloodstream infections. Also, the number and incidence of these infections caused by ESBL-producing *E. coli* increased and were highest in ≥ 60-year-olds. Simultaneous resistance to fluoroquinolones was high (nearly 80% in blood and 70% in urine).

**What are the implications of your findings for public health?**

When drawing up antimicrobial treatment guidelines, differences between infection types, age group, and sex should be considered. To focus prevention and control efforts, further research on sources of ESBL-producing *E. coli* is urgently needed.

## Introduction

*Escherichia coli* is the main cause of urinary tract infections (UTIs) and bacteraemias (blood stream infections; BSI) in high-income countries, causing substantial and increasing disease burden especially among elderly people [1-3]. Urinary tract infections, as one of the most common bacterial infections, are the leading source of *E. coli* BSI accounting for more than 50% of cases in high-income countries during 2007–2018 [1]. Several reports and studies published from high-income countries during 2020–2021 show that *E. coli* isolates obtained from BSIs and UTIs are becoming increasingly resistant to extended-spectrum β-lactams [4-8]. Furthermore, the emerging multidrug-resistant *E. coli* strains, such as extended-spectrum β-lactamase (ESBL) producing *E. coli*, are more challenging to treat and confer a higher risk of BSI and death [9].

According to the European Antimicrobial Resistance Surveillance Network (EARS-Net) report in 2019 [10], there was a small but significant increase in third-generation cephalosporin (3GC) resistance among invasive *E. coli* isolates during 2015–2019 in the countries of the European Union (EU) and European Economic Area (EEA). However, while this increasing trend was no longer observed in the report from 2020 [11], resistance was still high at 14.9%. In addition, considerable differences between countries were observed, with the percentage of resistant isolates ranging from 5.8 to 41.4 [11].

To our knowledge, nationwide population-based studies describing trends in the prevalence of ESBL-producing *E. coli*, juxtaposing urinary and blood isolates, and covering different age groups and sexes are scarce. In one study from Sweden, the proportion of ESBL-producing *E. coli* isolates, including cases detected by screening, was reported to have steadily increased among both sexes and within all age groups during 2007–2011 [12].

This study aims to investigate the proportions of ESBL-producing *E. coli* isolates and simultaneous resistance to fluoroquinolones among urine and blood *E. coli* isolates collected in Finland during 2008–2019. We also aim to characterise trends within different age groups and between sexes during this study period.

## Methods

### Surveillance data

The national Finres database [13], operated by the Finnish Institute for Health and Welfare (THL), contains antimicrobial susceptibility test results of 20 common clinically important bacteria under surveillance in Finland such as *E. coli*, *Staphylococcus aureus, and Streptococcus pneumoniae*. Data are entered annually for each bacterial species and only the first isolate with a susceptibility test result per sample type and patient is reported to this database. Information reported includes: (i) bacteria name; (ii) susceptibility test results for certain antimicrobials (disc diffusion, minimum inhibitory concentration (MIC), and/or interpretation of the test result); (iii) confirmed resistance mechanism for extended-spectrum β-lactams, meticillin and vancomycin; (iv) age and sex (male or female) and (v) date and type of specimen. 

These results are reported annually by all Finnish clinical microbiology laboratories that analyse blood and/or urine cultures, covering all healthcare districts in Finland. Reporting is mandatory. During 2008–2019, the annual number of laboratories reporting on blood cultures varied between 17 and 20 and the number of those reporting on urine cultures between 19 and 23. The Finres database covered approximately 98% of blood [8] and 96% of urine culture isolates sampled in Finland during the study period. All participating laboratories belong to the Finnish Study Group for Antimicrobial Resistance (FiRe), a clinical microbiology laboratory network. They are also government licenced, participate in international and national external quality assessment programmes as well as in the EARS-Net, and provide high quality laboratory services.

In each laboratory, the clinical specimens were cultured on appropriate growth media and bacterial growth was isolated and identified using standard methods such as biochemical tests, chromogenic culture medium, and/or matrix-assisted laser desorption ionisation-time of flight mass spectrometry (MALDI-TOF MS). Antimicrobial susceptibility tests including phenotypical ESBL screening and confirmation were performed and interpreted according to the Clinical and Laboratory Standard Institute (CLSI) guidelines [14] during 2008–2010 and according to the European Committee on Antimicrobial Susceptibility Testing (EUCAST) guidelines [15] during 2011–2019.

### Analysis and statistics

We calculated the annual proportions of ESBL-producing *E. coli* isolates from all urine and blood *E. coli* isolates for different sexes and age groups, expressed as percentage. In addition, we calculated the annual proportion of fluoroquinolone resistant ESBL-producing *E. coli* isolates, defined as ESBL-producing and resistant to moxifloxacin, levofloxacin, ciprofloxacin, and/or norfloxacin. We also calculated the annual incidences of these resistance profiles per 100,000 inhabitants. To compare observed trends over time and between age groups and sex, we applied a binomial regression model with log link and with or without Newey–West standard errors, which takes into account the possible autocorrelation conditional on the chosen trend. For average annual increases (AAIs) and trends, we calculated 95% compatibility intervals (95% CI) and p values, p values of < 0.05 were considered statistically significant. Data were analysed using SPSS Statistics 25 (IBM, .ibm.com) and Stata 17.0 (StataCorp LLC, .stata.com).

## Results

During 2008–2019, a total of 2,261,875 bacterial isolates from urine cultures and 108,952 from blood cultures were identified in the Finres database. The annual number of these isolates increased from 180,767 in 2008 to 188,342 in 2019 for urine cultures and from 6,746 in 2008 to 12,081 in 2019 for blood cultures.

Of these isolates, 1,568,488 (69.3%) urine culture isolates and 47,927 (44.0%) blood culture isolates were identified as *E. coli*. For all *E. coli* isolates, information on their ESBL status was available.

In addition, susceptibility test results for at least one fluoroquinolone were available for 96.0% (1,506,363/1,568,488) of the urine and 99.3% (47,611/47,927) of the blood *E. coli* isolates. For the urine *E. coli* isolates, the most common fluoroquinolone tested for resistance during 2008–2011 was norfloxacin (64.4% of fluoroquinolone-tested isolates; 298,155/463,093) and during 2012–2019 ciprofloxacin (78.0%; 813,604/1,043,270). For blood *E. coli* isolates, ciprofloxacin accounted for 62.7% (29,870/47,611) of the fluoroquinolone-tested isolates during the study period.

Of the urine *E. coli* isolates, 90.0% (1,411,644/1,568,488) were from females, 10.0% (156,842/1,568,488) from males, and two isolates were from individuals of unknown sex. Of the blood *E. coli* isolates, 61.2% (29,326/47,927) were from females and 38.8% (18,601/47,927) from males. The proportion of urine and blood *E. coli* isolates from patients aged ≥ 60 years were 65.3% (1,024,419/1,568,488) and 81.6% (39,131/47,927), respectively.

**Figure 1:**
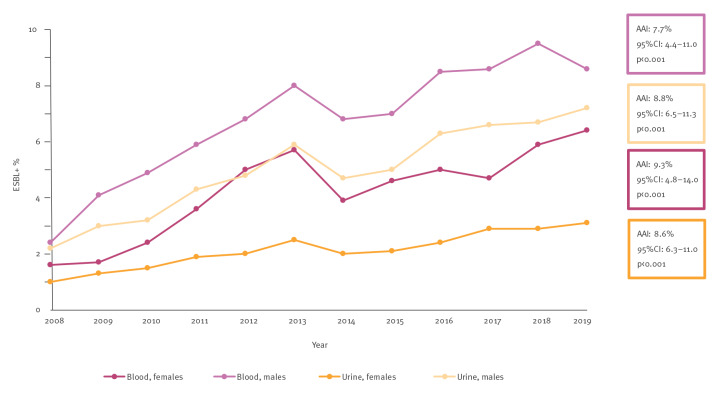
The annual proportion of extended-spectrum β-lactamase-producing *Escherichia coli* within urine and blood *E. coli* isolates among males and females, Finland, 2008–2019

AAI: average annual increase; CI: compatibility interval; ESBL+ : isolate positive for extended-spectrum β-lactamase-producing *Escherichia coli*.

During the study period, the annual proportion of ESBL-producing *E. coli* was consistently higher in blood than in urine *E. coli* isolates among both sexes (Figure 1). Moreover, it was consistently higher among males than females within both sample types (Figure 1). Importantly, a significant increasing trend in these annual proportions was observed as shown in Figure 1 and Supplementary Table S1 − annual proportions of ESBL-producing *E. coli* in blood and urine *E. coli* isolates. In blood isolates, the annual proportion of ESBL-producing *E. coli* more than tripled over the 12 years, among males from 2.4% (23/966) in 2008 to 8.6% (190/2,197) in 2019 (AAI: 7.7%; 95% CI: 4.4–11.0%, p < 0.01) and among females from 1.6% (28/1,806) to 6.4% (207/3,218) (AAI: 9.3%; 95% CI: 4.8–14.0%, p < 0.01). A similar increase was observed in urine isolates, among males from 2.2% (239/10,806) in 2008 to 7.2% (1,098/15,297) in 2019 (AAI: 8.8%; 95% CI: 6.5–11.3%, p < 0.01) and among females from 1.0% (1,045/108,390) to 3.1% (3,717/120,671) (AAI: 8.6%; 95% CI: 6.3–11.0%, p < 0.01).

**Figure 2:**
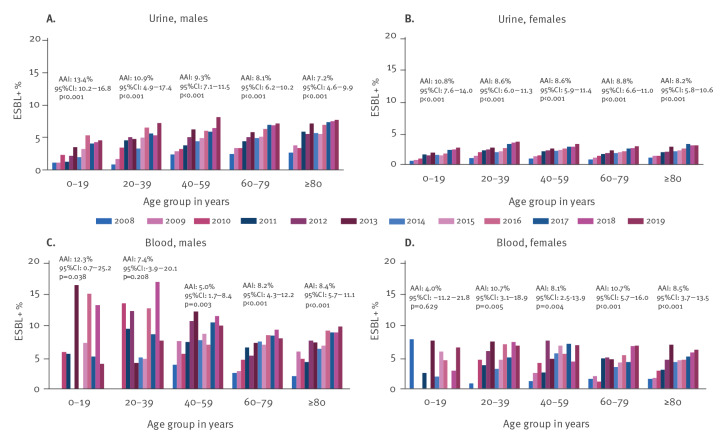
The annual proportion of extended-spectrum β-lactamase-producing *Escherichia coli* within urine *E. coli* isolates among (A) males and (B) females and within blood *E. coli* isolates among (C) males and (D) females stratified by age group, Finland, 2008–2019

AAI: average annual increase; CI: compatibility interval; ESBL+ : isolate positive for extended-spectrum β-lactamase-producing *Escherichia coli*.

In addition, similar significant increasing trends were observed within all age groups in both sample types (Figure 2 and Supplementary Table S1), except for blood isolates from males aged 20–39 and females aged 0–19 years. However, the annual proportions were higher in all male age groups compared with females in both sample types. Of note, the annual proportions and AAIs were similar between all age groups in both males and females.

**Figure 3:**
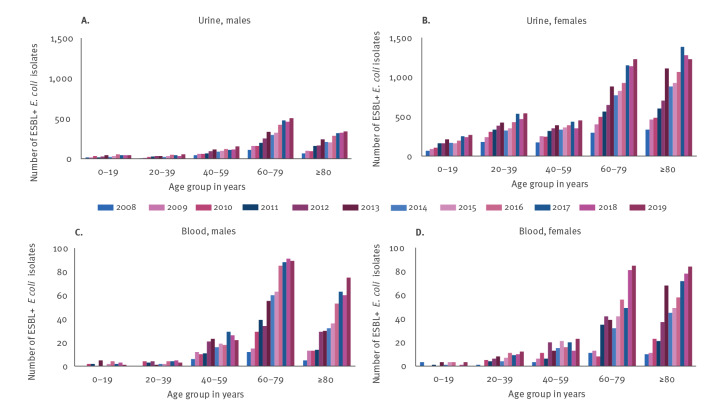
The annual number of extended-spectrum β-lactamase-producing *Escherichia coli* isolates within urine cultures among (A) males and (B) females and within blood cultures among (C) males and (D) females stratified by age group, Finland, 2008–2019

The majority of ESBL-producing *E. coli* urine (67.5%, 25,964/38,459) and blood isolates (80.2%, 2,132/2,660) were observed in the two oldest age groups (60–79 and ≥ 80 years) among both sexes and both sample types (Figure 3 and Supplementary Table S2 – annual numbers and incidences of ESBL-producing *E. coli* in blood and urine *E. coli* isolates). In urine cultures, the annual numbers of ESBL-producing *E. coli* isolates were considerably higher among all female age groups than males, but this was not observed in blood cultures.

ESBL+ : isolate positive for extended-spectrum β-lactamase-producing *Escherichia coli*.

**Figure 4:**
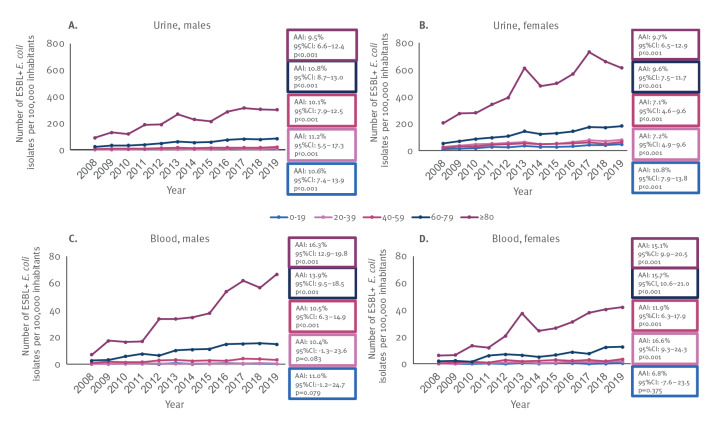
The annual incidence of extended-spectrum β-lactamase-producing *Escherichia coli* (numbers per 100,000 inhabitants) within urine cultures among (A) males and (B) females and within blood cultures among (C) males and (D) females stratified by age group, Finland, 2008–2019

AAI: average annual increase; CI: compatibility interval; ESBL+ : isolate positive for extended-spectrum β-lactamase-producing *Escherichia coli*.

The annual incidence of ESBL-producing *E. coli* was highest in the two oldest age groups (60–79 and ≥ 80 years) among both sexes in both urine and blood *E. coli* isolates, and in the ≥ 80 years age group it was higher than in 60–79 years age group (Figure 4). In urine cultures for the ≥ 80 years age group, the annual incidence of ESBL-producing *E. coli* was around two times higher among females than males. On the contrary, in blood cultures, the incidence was higher among males than females. Finally, the annual incidence of ESBL-producing *E. coli* increased significantly among all age groups within both sample types during 2008–2019, except for blood isolates from males and females aged 0–19 and males aged 20–39 years (Figure 4 and Supplementary Table S2).

**Figure 5:**
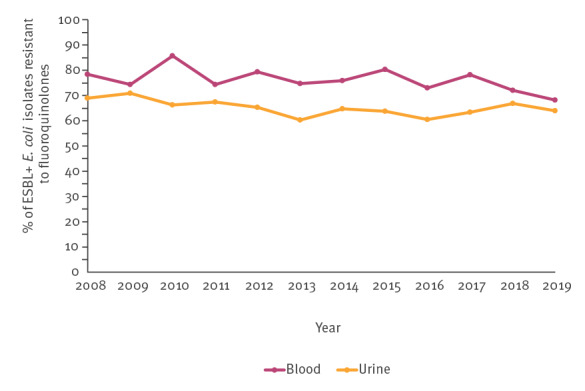
The annual proportion of fluoroquinolone resistance among extended-spectrum β-lactamase-producing *Escherichia coli* isolates from urine and blood, Finland, 2008–2019

In 2008, nearly 80% (40/51) of ESBL-producing *E. coli* blood isolates and 70% (885/1,284) of ESBL-producing *E. coli* urine isolates were resistant to fluoroquinolones (Figure 5). These percentages remained stable during the study period (Figure 5).

ESBL+ : isolate positive for extended-spectrum β-lactamase-producing *Escherichia coli*.

Of note, *E. coli* isolates from urine and blood cultures that were resistant or had intermediate susceptibility to meropenem and/or imipenem were rare during the study period (68 urine isolates, 0.004%, range by year: 2–16 and 13 blood isolates, 0.027%, range by year: 0–3).

## Discussion

Our study based on nationwide susceptibility testing results indicates that the annual proportion of ESBL-producing *E. coli* in both urine and blood *E. coli* isolates significantly increased during 2008–2019 in Finland. This increase was similar in all age groups regardless of sex. Moreover, the proportions of ESBL-producing *E. coli* were consistently higher among males than females in both blood and urine isolates.

Similar increasing trends in the proportion of ESBL-producing *E. coli* among clinical *E. coli* isolates have been observed worldwide in different studies [2,16,17] and surveillance reports [4-8,10]. However, comparing the results, particularly the annual proportions, is challenging due to differences in clinical diagnostic activity and surveillance methods such as different isolate reporting patterns and differences in reporting extended-spectrum β-lactam resistance [18]. Moreover, studies and surveillance reports differ in how age group, sex, sample type (urine/blood, clinical/screening), and healthcare vs community association are reported as well as how the annual incidences in demographic age groups have been analysed.

For *E. coli* blood isolates, the observed increasing trends in ESBL-producing *E. coli* were similar to reported trends in 3GC resistance among invasive *E. coli* isolates in other EU/EEA countries between 2008 and 2019 [19]. However, according to the EARS-Net dataset, the absolute proportion of 3GC resistance in Finland was approximately half (7.8% in 2019) of the average for EU/EEA countries (15.1% in 2019, range: 6.2–38.6%) [10,19], but similar to other Nordic countries including Sweden, Norway, Denmark, and Iceland (average in 2019: 7.1%; range: 6.2–7.8%) and the Netherlands (7.5% in 2019) [19]. For urine *E. coli* isolates, both the trends and annual proportions of ESBL-producing *E. coli* we observed in this study were similar to those reported in other Nordic countries [5-7]. In addition, in the United States (US), a similar yearly increase of 7.7% was reported for ESBL-producing *E. coli* among urine *E. coli* isolates from adult and adolescent female outpatients during 2011–2019 [20]. However, the annual proportion in the US (7.3% in 2019) was approximately double the proportion among female in- and outpatients in Finland (3.1% in 2019).

It is important to note that 90% of all observed ESBL-producing *E. coli* urine isolates were from females. However, this percentage was 61% for ESBL-producing *E. coli* blood isolates. As only the first *E. coli* isolate with a susceptibility test result per sample type and patient is reported to the Finres database, there should not be any duplicates in our study. This indicates that UTIs caused by ESBL-producing *E. coli* were overall far more common among females. However, among males, the UTIs were presumably more often complicated resulting in BSI as more than half of *E. coli* BSIs are considered to derive from the urinary tract in high-income countries [1]. In a previous study conducted in San Francisco, US, during 2014–2020, male sex and advanced age were associated with a higher proportion of ESBL-producing *E. coli* among *E. coli* bacteriuria episodes [16], which was also observed in our study. However, we observed notably higher proportions of ESBL-producing *E. coli* within *E. coli* blood isolates among males than among females. This may be related to urological procedures and frequent urinary catheterisation, which have previously been reported as risk factors for community-onset ESBL-producing *E. coli* BSI and other infections [21,22]. Also, due to the increased risk of complications in males, their UTIs are likely more often treated with extended-spectrum antimicrobials than females’ UTIs.

Perhaps the most concerning finding of our study was the constantly increasing proportion of ESBL-producing *E. coli* within blood *E. coli* isolates, which is also in line with surveillance reports from other Nordic countries [5-7]. According to our study, the annual number of all blood isolates reported to the Finres database nearly doubled during the study period and *E. coli* accounted for 44.0% of these isolates. This is in line with a previous Finnish study based on different surveillance data from 2004–2018 [3]. The increasing proportion of ESBL-producing *E. coli*, especially in blood *E. coli* isolates, should be monitored carefully to prevent empirical treatment failures since nearly 9–10% of recent blood *E. coli* isolates from males were ESBL-producing, and intravenous cefuroxime is the drug of choice for empirical treatment of severe febrile UTI (e.g. pyelonephritis) and BSI in Finland [23]. Furthermore, the increasing proportion of ESBL-producing *E. coli* may result in increased use of carbapenems, particularly for severe infections, as nearly 80% of blood and 70% of urine ESBL-producing *E. coli* isolates were also resistant to fluoroquinolones. Fortunately, carbapenem-resistant *E. coli* isolates were extremely rare in Finland during the study period.

The majority of ESBL-producing *E. coli* isolates were observed in individuals aged ≥ 60 years. This demonstrates that the highest morbidity of ESBL-producing *E. coli* as a cause of UTI or BSI was in individuals aged ≥ 60 years. Similar results can also be derived from a Swedish surveillance report [5]. Concomitantly, our results demonstrated that the risk of acquiring an ESBL-producing *E. coli* UTI was greatest in females aged ≥ 80 years, the demographic group in which the incidence was highest. In contrast, males aged ≥ 80 years had the greatest risk of BSIs. Furthermore, it is important to note that both the morbidity and risk of ESBL-producing *E. coli* UTIs and BSIs also increased in most other age groups during the study period. This was also demonstrated in an earlier Swedish study, in which the number of ESBL-producing *E. coli* isolates were shown to increase steadily in males and females in all age groups during 2007–2011 [12]. These studies are not directly comparable, however, as the Swedish study included screening samples.

It is important to note that differences in national or local clinical guidelines and practices in collecting bacterial culture samples may influence the observed resistance proportions. For example, if clinical samples are mostly collected for diagnosis and management of more complicated UTIs, such as pyelonephritis, this may lead to an overestimation of ESBL-producing *E. coli* proportions since cystitis is the most common UTI. In Finland, the diagnosis and management of acute cystitis (uncomplicated UTI) can be based on structured interviews in otherwise healthy women aged 18–65 years according to the Finnish current care guidelines [23]. If empirical treatment fails in this age group, urinary cultures are requested [23]. In turn, urinary cultures are requested for diagnosis and management of all other UTI cases (complicated UTIs such as pyelonephritis, urosepsis, and UTIs of males, children, and women over 65 years of age) in Finland [23]. In our data, this may have led to a slight overestimation in the proportion of ESBL-producing *E. coli* in UTIs of women aged 18–65 years compared with the other demographic groups. In addition, in clinical practice, despite the current care guidelines, some of the complicated UTIs may be treated empirically without culturing the urine, or the urinary cultures may be taken after the initiation of antimicrobial treatment lowering the sensitivity of finding the pathogen [24], which may have led to a slight overestimation in the proportion of ESBL-producing *E. coli* in UTIs in our data.

Our study has some limitations. First, we could not differentiate community- and healthcare-associated isolates and we had no data on the molecular epidemiology of these isolates. A previous study has shown CTX-M to be the most common resistance mechanism (79%) in nosocomial BSIs caused by ESBL-producing *E. coli* and *Klebsiella pneumoniae* in Finland during 1999–2010 [25]. In addition, the rates of nosocomial BSIs caused by 3GC-resistant *E. coli* and *K. pneumoniae* increased more sharply than the overall national rates in Finland [25]. Second, within the dataset, blood and urine isolates could not be matched so information on which of the urine isolates caused BSIs is unknown. Third, there was no information about severity and clinical outcome of the infections or data on underlying medical conditions and surgical or other procedures in the patients.

Finally, to slow down the increasing trend of ESBL-producing *E. coli* UTIs and BSIs in Finland, more research on source attribution for ESBL-producing *E. coli* is needed. International travel, especially to high prevalence countries, has shown to be associated with acquisition of ESBL-producing *Enterobacteriaceae* [26,27] and development of community-onset ESBL-producing *E. coli* infections [28]. In a publication from 2015, where Finnish travellers who had visited destinations outside the Nordic countries were investigated, around 20% of them were reported to have become colonised by ESBL-producing *Enterobacteriaceae. *In that study, travellers’ diarrhoea (TD) and antimicrobial use for TD were found as independent risk factors for colonisation [29].

The probability of onward transmission of ESBL-producing *Enterobacteriaceae* within households of travellers has been estimated to be 12% [30]. However, the extent of further spread in healthcare facilities, particularly in nursing homes, is not known. In addition, antimicrobial use and the presence of ESBL-producing *E. coli* in livestock and livestock products may also contribute. However, the latter is probably not the case in Finland as no clear evidence of genetic overlap between human and animal, food, or environmental ESBL-producing *E. coli* isolates was observed in a study from 2022 [31]. Antimicrobial use, in turn, is a well-recognised driver of antimicrobial resistance (AMR). In Finland, the annual total consumption of antimicrobials, including fluoroquinolones, for treating patients decreased during 2013–2020 [32]. During the same period, 3GC consumption was stable, although overall higher than in other Nordic countries [32]. However, the proportion of ESBL-producing *E. coli* increased during the same period in Finland according to our study. Furthermore, in Norway, multidrug-resistant clonal complex (CC)131 *E. coli* isolates significantly increased during 2002–2017 despite low antibiotic use [33]. Hence, the important question is whether the decrease in antimicrobial consumption was too limited, especially among patients in certain risk groups, such as patients in nursing homes and other long-term care facilities and hospital wards, to prevent the increase in ESBL-producing *E. coli* isolates.

## Conclusion

Our nationwide study demonstrates that the highest risk and greatest morbidity of ESBL-producing *E. coli* UTIs and BSIs in Finland are in patients aged ≥ 60 years, in whom *E. coli* causes major and increasing morbidity. Furthermore, the proportion of ESBL-producing *E. coli* in urine and blood *E. coli* isolates increased during the study period being constantly higher among males than females. As most ESBL-producing *E. coli* isolates are also resistant to fluoroquinolones, this increase may severely limit effective treatment options of severe infections such as pyelonephritis and urosepsis, ultimately leading to the increased use of carbapenems. These observed trends need to be carefully monitored, highlighting the importance of AMR surveillance. Moreover, the main drivers behind ESBL-producing *E. coli* acquisition in Finland require further investigation.
